# Polyploidy: A macromutational force pushing bioeconomic developments

**DOI:** 10.1073/pnas.2522065123

**Published:** 2026-05-26

**Authors:** Marlies K. R. Peeters, Yves Van de Peer

**Affiliations:** ^a^ Department of Plant Biotechnology and Bioinformatics, Ghent University, 9052 Ghent, Belgium; ^b^Flanders Research Institute for Agriculture, Fisheries and Food, Plant Sciences Unit, Melle 9090, Belgium; ^c^VIB Center for Plant Systems Biology, Ghent 9052, Belgium; ^d^ Centre for Microbial Ecology and Genomics, Department of Biochemistry, Genetics and Microbiology, University of Pretoria, Pretoria 0028, South Africa; ^e^ College of Horticulture, Academy for Advanced Interdisciplinary Studies, Nanjing Agricultural University, Nanjing 210095, China

**Keywords:** bioeconomy, genetic diversity, adaptability, metabolic capacity, morphological changes

## Abstract

Polyploidization, the consequence of genome doubling, is a macromutation that reshapes genomes, phenotypes, and ecological interactions. Polyploidization often results in novel phenotypes, including alterations in size, physiology, biochemistry, and enhanced stress tolerance. Here, we discuss how strategically leveraging polyploidy can provide significant advancements within the modern bioeconomy committed to reducing our ecological footprint through the sustainable production and use of biological resources. The bioeconomy spans diverse sectors, including agriculture, health sciences, and biotechnology. By elucidating and leveraging the immediate, or short-term, effects of polyploidization, such as harnessing genetic diversity, extensive biomass production, diversification of metabolites, and improved stress resilience, we highlight how this process unlocks vast, underexplored bioeconomic opportunities. This includes accelerating the exploration of new breeding techniques, speeding up the domestication of new local varieties or medicinal plants, and offering possibilities for improved biofuel production, bioremediation strategies, therapies, and production and discovery of bioactive compounds. The multilayered effects of polyploidization shared across sectors can foster interdisciplinary exchange and are essential for advancing toward a more sustainable bioeconomy.

Whole-genome duplication (WGD), leading to polyploidy, is a widespread phenomenon that has been studied for over 100 y (1). Polyploidy can occur naturally or can be induced, affecting cells, tissues, or entire organisms. Although polyploidy is often detrimental due to genomic instability, mitotic and meiotic abnormalities, and minority cytotype exclusion (2), polyploid organisms exist everywhere, but particularly in plants (3, 4). Ancient polyploidy events coincide with the origin and diversification of major phylogenetic lineages, including vertebrates, fishes, and flowering plants, and within flowering plants, core eudicots, monocots, orchids, grasses, composites, and legumes (5), suggesting a role for WGD in phenotypic diversity, with a subsequent facilitating role in speciation (6, 7). Moreso, ancient polyploidy events seem to correlate with periods of climate change (8).

Polyploidization is frequently associated with novel phenotypes. Doubling the DNA content can shift gene expression, metabolism, and epigenetic modelling, with major consequences for development and physiological responses (Box 1) (9–11). These polyploidy-linked responses have long been exploited in agriculture, because of their desirable agronomic value, such as enhanced size and stress tolerance, resulting in more productive and sustainable crop varieties. Although most polyploidy applications so far have been framed within the so-called green biotechnology and bioeconomy, we here describe how short-term effects of polyploidization are increasingly exploited across blue, white, and red bioeconomies (Box 2, Fig. 1, and *SI Appendix*, Fig. S1).

**Fig. 1.**
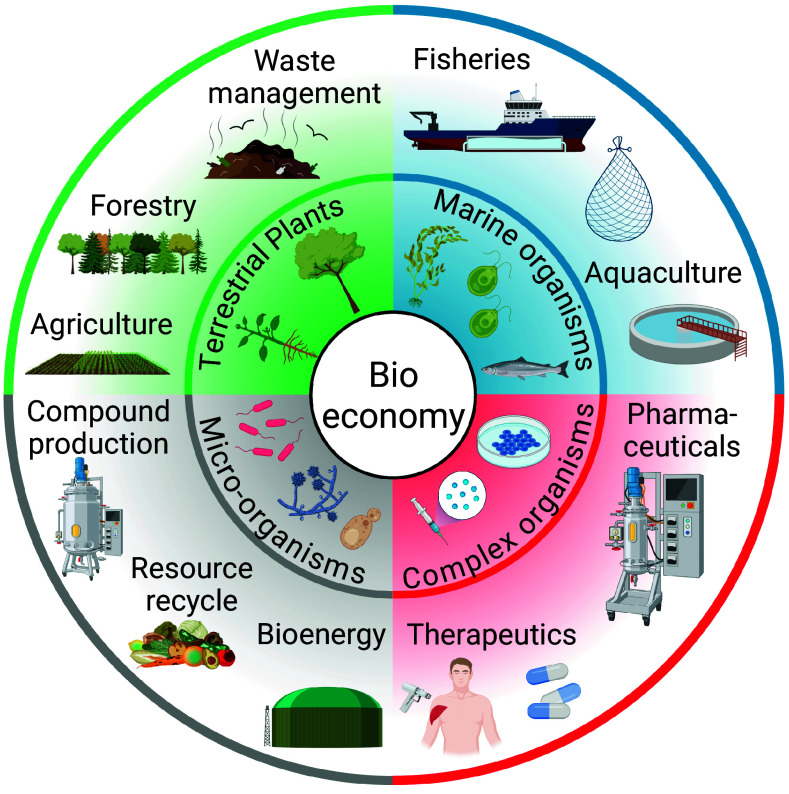
Overview of different pillars of bioeconomy with the most prominent organisms and the sectors. Created with BioRender.com/cpbnyjf.

A modern and sustainable bioeconomy is widely viewed as a route to address climate change, insufficient food production, pollution and polluting energy generation, and biodiversity loss (12). This transition from fossil-based to bio-based products and energy aims to support a carbon-low society, sustainable food production, and bio-based resources for materials, energy, and services. Because it spans sectors from agriculture, ecology, and forestry, to waste management, bioenergy and biofuels, pharmaceuticals and health care, the bioeconomy has a central role to reach the sustainability goals postulated in 2015 in the European Green deal, aiming to transition into a cleaner, healthier, and more resource-efficient economy while protecting ecosystems and addressing social and regional inequalities (12, 13). Here, we summarize applications by illustrating selected examples in which polyploidization leverages the goals of climate neutrality, investment in renewable energy, circular economy, ecosystems, and pollution reduction (Box 3 and Fig. 2).

**Fig. 2.**
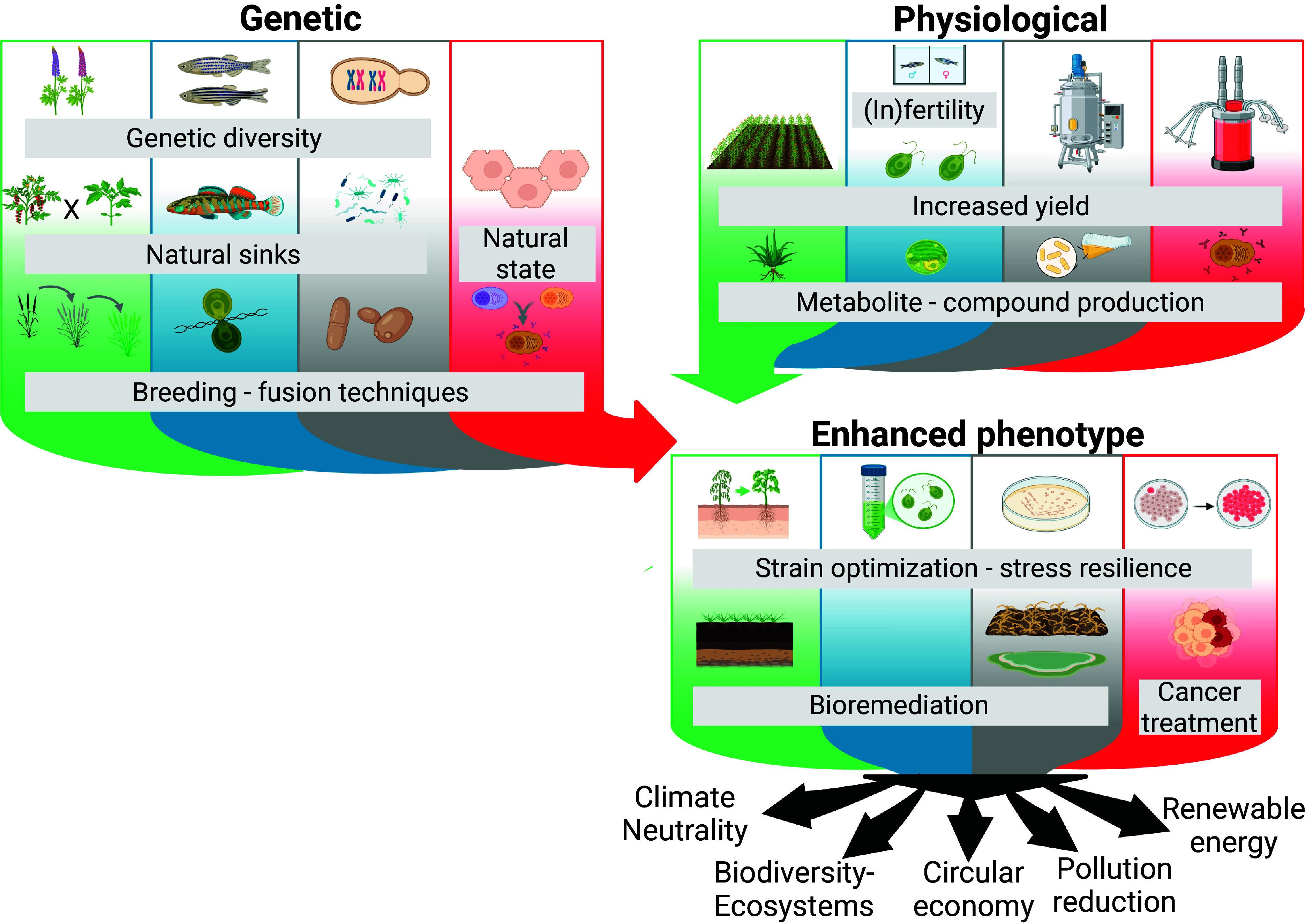
Mechanistic roadmap of polyploidization effects and their application in bioeconomy as plausible pathways for policy-relevant targets. Both genetic and physiological changes lead to enhanced phenotypes, which can be employed to reach several goals of the European Green Deal. Created with BioRender.com/t7qsb5b.

Box 1. The polyploidy continuum.

Polyploidy can be viewed as a continuum, with autopolyploidy and allopolyploidy as conceptual extremes (14). Autopolyploids arise through DNA replication or cell division errors, leading to the duplication of the same set of chromosomes within an organism, and thus carrying multiple chromosome sets from a single species with low levels of diversity. By contrast, allopolyploids form through hybridization between different species, often followed by chromosome doubling, creating highly divergent sets of chromosomes and thus differentiated parental subgenomes. Many polyploids can be situated along the continuum as segmental or intermediate polyploids, where partially diverged subgenomes can still recombine and different loci or chromosomes exhibit mixtures of auto- and allo-like pairing and inheritance. Recognizing this continuum is crucial for interpreting its evolutionary consequences and for designing effective breeding and engineering strategies.

Box 2. Different bioeconomies

Parallel to the classification used in biotechnology (15), different branches of the bioeconomy are color coded based on their prime biological resource. Green bioeconomy focuses on sustainable use of terrestrial biological resources (e.g., crops, forests, soils, and ecosystems), to produce food, materials, chemicals and energy in low-carbon, resource-efficient ways, while reducing greenhouse gas emissions, and conserving biodiversity across agriculture, forestry, and waste management. Blue bioeconomy focuses on sustainable use of aquatic resources, (e.g., fisheries, aquaculture) to generate food, feed, materials, and high-value products while safeguarding aquatic ecosystems. White bioeconomy refers to industrial biotechnology, using microorganisms and enzymes to produce biofuels, bioplastics, and other products from renewable biological resources with reduced environmental footprints. Red bioeconomy encompasses health and medicine biotechnology, including biopharmaceuticals, vaccines, advanced therapeutics, and regenerative medicine to improve disease prevention, diagnosis, and treatment.

Box 3. Stress tolerance of polyploids in bioeconomy

Polyploidization (genome doubling) is a macromutation that significantly impacts cellular and organismal biology, particularly in plants, yet its broader potential for the bioeconomy remains underexploited. By increasing genetic diversity and shifting gene expression, metabolism, and morphology, polyploidy can enhance stress tolerance to abiotic and biotic challenges, a trait relevant to food security, bioenergy, green chemistry, and health. While widely used in crop improvement, polyploidization applications in aquaculture, microbial bioprocesses, bioremediation, and biopharmaceutical production remain fragmented and discipline specific. Synthesizing these immediate effects and applications across green, blue, white, and red bioeconomies, highlights opportunities where polyploidization could drive innovation toward a sustainable bioeconomy. Recognizing polyploidization and the bioeconomic potential of its enhanced stress tolerance as a shared mechanism across sectors can bridge disciplinary boundaries and guide investments toward climate-smart, health-relevant biotechnologies.

## Green Bioeconomy

### Unlocking Genetic Diversity in Plants.

Green bioeconomy aims to sustainably improve terrestrial crops and agroecosystems, harnessing genetic and genomic tools to enhance productivity, resilience, and ecosystem services. Within this framework, genome doubling has long expanded genetic diversity in breeding germplasms and remains a major crop improvement strategy (16). Monoculture and intensive selection for high productivity have narrowed the genetic base of many crops, increasing vulnerability to disease and environmental change (17–19). Thus, broadening diversity, within varieties, across crops, and through improved utilization of underutilized crops, by sustainably introducing genetic variation, is a key route to strengthen global food production (19). For example, polyploid germplasm enabled improved fresh herbage (16) and promising phenotypic variation of synthetic allohexaploid *Brassica* (20). In horticulture, polyploid germplasm has supported breeding of fruitless, low-maintenance ornamental *Melastoma* shrub cultivars (21), blue tropical and temperate *Hibiscus* species (Malvaceae) (22), and cold resilient *Lagerstroemia indica* trees to enlarge ornamental range (23).

However, polyploidization alone rarely rebuilds sufficient diversity within germplasms, and access to the genetic pool of wild relatives is becoming increasingly pressing. Polyploidization can act as a genetic bridge between cultivated populations and otherwise inaccessible wild genetic pools. Theoretical models predict gene flow between mixed-ploidy populations (24) and natural introgression from wild relatives has been documented in the Yangambi coffee breeding collection (25). In potato, tetraploid cultivars were hybridized with wild diploids via unreduced gametes to introgress novel genetic variation (26, 27), while interspecific hybrids between wild and cultivated peanut (*Arachis*) yielded promising plants with improved bacterial wilt resilience (28). Thus, polyploidization can facilitate transfer of beneficial genes, hidden in the genetic pool of wild relatives, into domesticated crops to strengthen genetic diversity.

A further underexploited reservoir lies in native and underutilized species. Current breeding has largely focused on improving existing crops and only limited resources were devoted to investigating the potential of local agriculture, both short-chain local production systems and native, yet undomesticated plant species (29). Directed breeding programs using local polyploid cultivars could enhance food security in agriculturally difficult regions while supporting a bioecology vision that sustains local varieties and ecosystems (29). Several underutilized crops offer high potential for commercial use, diversified diets, income for local communities, and positive effects on soil health. Although domestication faces biological barriers (29), polyploidization can help to overcome constraints and optimize agricultural values, boosting local agriculture. For example, triploid Ethiopian banana cultivars (*Ensete ventricosum*), a primary food source for over 20 million people, outyielded diploids due to its enlarged nutritious pseudostems (30).

Beyond generating variation and enabling introgression, altering ploidy supports new breeding techniques, such as doubled haploid (DH) breeding. Artificially doubling a haploid genome, produced via androgenesis or gynogenesis, shortens breeding cycles, improves breeding efficiency, and fixates genetic traits by creating homozygous lines within one generation (31). DH breeding is already used in maize, barley, and wheat, but could accelerate breeding of other economically relevant and/or sustainable crops with slow traditional breeding cycles, such as cassava and poplar (32, 33). It can further help circumvent interspecific sterility mediated by multiple sterile hybrid genes (34). For example, Asian and African rice (*Oryza sativa*, resp. *Oryzaglaberrima*) were crossed at the tetraploid level, and subsequent androgenesis generated genetically balanced interspecific diploid hybrids carrying fixed traits of both parents (34). By modulating hybrid ploidy throughout crossing schemes, this method can yield new interspecific varieties harboring superior parental traits. Additionally, DH breeding allows the introduction of resistance genes via inducer lines, producing genetically modified plants without remaining transgenic elements (31), potentially easing public concerns over gene editing methods. Yet, DH breeding is only limitedly employed, due to the lack of appropriate routine protocols with sufficient viable embryos and subsequent plants (35). Additionally, because DH production instantaneously fixes the entire genome in a homozygous state, it can be particularly inefficient in germplasm with high mutational or genetic load (e.g., landraces, highly mutagenized populations), where exposed recessive deleterious alleles can reduce viability or fertility (36). Further investigation of DH could advance future bioeconomic crop development, both by accelerating breeding and introducing modified resistance genes.

Despite well-documented advantages of genetic diversity, a paradigm exists between the need to diversify traits and the strict requirements for registering new varieties defined by the DUS criteria (distinctness, uniformity, and stability), which emphasize morphological uniformity (37). This framework penalizes genetically complex and phenotypically variable crops, including polyploids and outbreeding species, which struggle to meet these outdated morphological assessments. This underscores the need to reform current registration systems to better reflect contemporary climate-resilient plant breeding goals (37). To support these breeding strategies, a genomics-enabled registration approach integrating molecular tools has been proposed to better accommodate genetically diverse crops (38).

### Exploit Gigas Effect for Sustainable Biomass and Biofuel Production.

The increase of cell volume and/or organ size (i.e., gigas effect) is a long-known consequence of polyploidization and has been extensively exploited to boost yield and enlarge ornamentals. Major crops, including maize, sugarcane, sorghum, and forage grasses, collectively occupying ~17% of global land area (39), have undergone domestication and polyploidization, and show long-term genomic stability, indicating that polyploid-derived benefits can become durable (40). However, phenotypic expansion through polyploidization is underexplored in some economically important crops like maize, and successful tetraploid maize varieties with good agronomic performance are only recently being registered (41).

Beyond food and feed, yield gains can also increase biological resources that can be processed and upcycled into new value chains, including biofuels, as envisioned bioeconomic transition. Biofuels can reduce net carbon emissions, but their sustainability depends on maximizing biomass with minimal inputs (42). Because starch-based biofuels (first-generation) compete with food production, efforts shifted to lignocellulosic materials, waste, and nonfood crops (second-generation), and algae (third-generation) (43). Cultivating orphan lignocellulose crops that can grow on marginal lands could supply biomass for cellulosic ethanol (42), but the resistance of cell wall components to degradation (lignocellulose recalcitrance) enforces extensive pretreatments that lower efficiency. Polyploidization can address these constraints, by increasing biomass and modifying cell wall properties. Polyploids of *Miscanthus* (44) and willow (45) showed enhanced biomass, while altered cell wall composition and reduced cell wall-to-cell volume ratios in polyploid cells can improve processability (46). Indeed, straw from tetraploid potato generated high bioethanol yields using mild pretreatment and additionally reduced agricultural waste by valorizing all parts of the plant (47). Sugarcane is another promising bioenergy crop due to its genetically diverse polyploid germplasm, suited to combine disease resistance and hardiness with high sugar contents and environmental growing flexibility in new varieties (48). The nonedible allohexaploid *Brassica* further showed improved phenotypic characteristics such as increased biomass, protein content, and glucosinolate content; but limited effects were observed for oil content and seed yield (20). Likewise, *Brassica carinata* is a suitable bioresource for biofuel, combining broad adaptability and high productivity under suboptimal conditions, rotation possibilities with maize due to winter hardiness and expansion of usable land via phytoremediation (49), aligning polyploid crop development with sustainable land use. Similar advantages exist for *Camelina sativa*, used for sustainable jets and rockets biofuel with only limited impact on engine performance (50). Because polyploidy-induced advantages vary across bioenergy crops and are mostly beneficial when vegetative biomass is the primary product (51), careful selection and evaluation remain essential for meeting Green deal goals and benefit from the untapped potential in polyploid bioenergy crops.

### Increased Diversity of Bioactive Plant Metabolites.

Polyploidy can diversify metabolic pathways by increasing genetic content and consequently, metabolite quantity, diversity, and chemical structure (52, 53). Much plant metabolic complexity likely arose via gene duplication, followed by sub- and neofunctionalization (54), and natural plant metabolites (e.g., morphine, steroids) remain central to drug discovery, both as models for designing new drugs and as therapeutics from natural sources. Indeed, medicinal plants are widely used in nonindustrialized societies because of affordability and availability (55), including pomegranate, valued for its astringent rind, and anis (*Agastache Mexicana*), exploited for tilianin with therapeutic potential in cardiovascular diseases (55, 56). Tetraploid pomegranates have been developed to assess fruit traits (57), but effects on the medically relevant rind remain unclear, whereas autotetraploid anise produced higher tilianin levels than its diploid progenitor (56). Yet, only a few studies have examined polyploidization in native medicinal plants. Given that ~80% of people in developing countries rely on medicinal plants (52), optimizing local varieties via polyploidization could increase phytochemical yields and strengthen small-scale, locally rooted production systems.

Despite growing markets and therapeutic value, relatively few medicinal plants are cultivated, and few compounds can be produced at industrial scale (53, 58). Expanding genomic resources of medicinal plants now enables more targeted high-yield breeding and metabolic engineering (58). These resources combined with polyploidization-driven metabolic diversification can broaden the spectrum of bioactive products and refine working mechanisms. For instance, WGD of *Eriobotrya japonica* (loquat) generated diverse triterpene chemotypes, anti-inflammatory compounds used in asthma medicine, across species and developmental stages, via differentially evolved duplicated gene pathways (59). Similarly, colchicine-induced polyploidization in *Stachy byzantina* improved the production of phytopharmaceuticals like linalool (60). Although polyploidization-driven enhanced secondary metabolite production is reported in a wide variety of medicinal plants, outcomes are not uniformly positive due to genome instability and variable outcomes of gene redundancy (53). Nevertheless, systemic exploration of polyploidization to diversify metabolic pathways could further expand the portfolio of bioactive plant compounds for both local healthcare and global pharmaceutical development.

### Stress Resilience: From Marginal Lands to Ecological Balance.

Another promising consequence of polyploidization is the emergence of novel phenotypic variation in newly formed polyploid (neopolyploid) populations, potentially enhancing tolerance to biotic and abiotic stresses across short- and long-term environmental change (8, 61, 62). Neopolyploids occur more frequently in human-disrupted and environmentally demanding habitats, highlighting their potential in climate-affected environments (10, 11). Indeed, climate change currently reshapes crop-growing conditions through extremes in temperature, water, radiation, and nutrients supply, causing substantial economic losses (63, 64), while breeders are at the same time tasked with developing crops for unknown, future environments. Among the numerous examples of polyploid-associated resilience are improved heat tolerance in tetraploid *Asparagus officinal* (65), enhanced cold tolerance in the Chinese medical tetraploid *Dendranthema nankingense* (66) and combined drought and cold tolerance in tetraploid ryegrasses (67). Additionally, polyploidization can also improve drought tolerance, water-use efficiency, and resources requirements in altered soils. For example, tetraploid rice shows increased salt tolerance via faster activation of stress-responsive genes in the jasmonic acid pathway via DNA hypomethylation (68), while octoploid broomcorn millet displayed stronger antioxidant capacity under salt stress, strengthening osmoregulation (69). However, diverse outcomes among natural neopolyploids highlight that polyploidy does not guarantee adaptive or demographic success (11) and deliberate polyploidization for bioeconomic applications should be accompanied by comprehensive phenotypic, ecological, and evolutionary assessment rather than assumed advantage.

Despite benefits in agriculture, polyploidy remains underutilized in forestry (70), although reforestation is central to climate mitigation and biodiversity restoration. For most economically valuable tree species, polyploidy effects remain largely untested, especially under field conditions, with only a few and sometimes contrasting observations (71). Long generation times likely hinder experimentation, but available evidence suggests that polyploidization could expand breeding possibilities, improve industrial traits, and enhance stress resilience, offering untapped opportunities to advance sustainable forestry and climate adaptation.

Increased resilience attributed to polyploid crops may also enable cultivation in previously unsuitable areas, or salinized soils caused by human activities, irrigation, and climate change (72). Niche expansion of economical crops optimizing land usage is illustrated by the cereal broomcorn millet adapted to grow in adverse conditions (73), and the allotetraploid *Brachypodium hybridium* exhibiting greater ecological flexibility and higher drought adaptation associated with higher photosynthetic capacity (74). Polyploidization may further influence the soil microbiome and restore soil health after pathogen accumulation and nutrient imbalances caused by intensive monoculture, complementing crop diversification and rotation (75). While reciprocal interactions between soil microbiomes and polyploid plant phenotypes have been proposed (76), evidence of polyploid plants altering the soil composition remains limited. For wheat varieties, ploidy levels explained variation in rhizosphere bacterial communities (77), while the diversity of bacterial communities increased around tetraploids of the invasive *Centaurea maculosa* (78). Further exploring soil-polyploid plant interactions can uncover previously overlooked sustainable opportunities for restoring soil health, expanding arable land, and sustaining productivity.

Polyploid organisms also offer underappreciated opportunities in wastewater treatment and sustainable water-resource management, which has become a priority in contemporary policy frameworks due to intensified freshwater scarcity (13). Aquatic plants can bioaccumulate pollutants and remove residual nutrients in energy-efficient and environmentally friendly way. Polyploid duckweeds are particularly promising due to rapid growth and broad environmental tolerance (79), illustrated by the often-better performance under abiotic stresses, including elevated metals, salinity, and urban wastewater conditions (80, 81). Polyploid terrestrial species may also support water recycling. For instance, tetraploid *Cenchrus ciliaris* grew when irrigated with treated wastewater (82), illustrating how polyploidy can strengthen and diversify plant-based wastewater valorization.

Beyond managed systems, polyploid stress tolerance and plasticity can enable colonization of habitats unsuitable for diploid ancestors (83). In kiwifruit (*Actinidia chinensis*), tetraploids occur at higher altitudes and in colder climates than diploids, reflecting niche differentiation by ploidy level (84). Neopolyploidy can further contribute to speciation in challenging environments, but neopolyploids may succeed and invade or fail (11, 85). Because polyploid survival frequently coincides with environmental upheaval (8), the adaptive capacity of polyploids may help natural populations evolve toward new niches and establish new ecological balances, maintaining biodiversity currently pressured by human activities.

### Blue Bioeconomy.

#### Polyploid germplasm and genetic innovation in aquaculture.

The blue bioeconomy, centered around sustainable utilization of aquatic biological resources to develop innovative products and services, is expanding rapidly. As in terrestrial agriculture, polyploidization offers opportunities to genetically improve breeding germplasm in commercial aquaculture. Given aquaculture’s potential to serve as an alternative source of animal protein, there is rising demand for accelerated growth and enhanced resistance to disease and environmental stress (86, 87). Tetraploid organisms serve as core germplasm to generate triploid hybrids, for example, in rainbow trout and Pacific oysters (88, 89). Triploid fish, which lack functional gonads, often display superior aquacultural traits including improved growth rates and yield. The absence of sexual maturation prevents diversion of fatty acids to gonadal development, thereby enhancing fillet quality and extending the productive lifespan (90). This sterility can further prevent mixing domesticated and wild populations (91). Within this framework, triploidy has long been applied in commercial aquaculture species such as rainbow trout and Atlantic salmon, using thermal or high-pressure shock to prevent introgression of genes into wild populations (92, 93). As investments in aquaculture explore transgenic fish to increased growth, disease resistance, better feed conversion efficiency, and digestion of land-based foods to improve food availability and reduce production costs (94), ploidy-based fertility control can prevent disturbing local wild populations, limiting introduction of transgenic genes into natural populations and help address public concerns about transgenic food supply. Manipulating fertility through ploidy offers thus a robust biocontainment strategy, inevitable to overcome the ecological concerns while benefitting from biotechnological advances.

Beyond artificially induced polyploids, naturally occurring polyploid populations represent valuable reservoirs of genetic diversity and promising candidates for valorization. Allotetraploid common carp, for instance, is globally distributed and thermally tolerant, with numerous locally adapted varieties. Its versatility makes it an ideal germplasm source for both production and ornamental purposes (87). Over the past decade, hybrid carp species spanning various ploidy levels have been developed to optimize aquacultural performance, positioning the common carp as a model organism for studying vertebrate hybridization (87). Likewise, ornamental allotetraploid goldfish (*Carassius auratus*) can serve as an example for how polyploidy facilitated broad phenotypical diversity throughout domestication, as recent genomic resources identified asymmetrical subgenomic evolution as a major contributor to phenotypic diversity currently observed (95, 96). Another promising example is *Schizothorax curvilabiatus*, a polyploid fish endemic to the Tibetan Plateau with significant ecological and economic potential (97). Initial steps toward its domestication have included the development of genomic resources, yet further investment is needed to fully harness its unique adaptations (87).

#### Polyploid algal resources for fuel and pharma.

Algae have now become key in the blue bioeconomy efforts (98). They are ideal alternative biomass resources for biofuels, due to their fast multiplication cycle, efficient energy conversion, and CO_2_ fixation, without competing for scarce fresh water or arable land used for food production (99). Like bioenergy crops, high biomass accumulation remains a prerequisite to be a sustainable resource stream for biofuels. In the microalga *Chlamydomonas,* doubled-genome strains demonstrated increased biomass accumulation and stress tolerance (100), while polyploid cultures of *Dunaliella salina* showed higher biomass production after phytohormone treatments (101). As in finfish aquaculture, triploid sterile algal lines are also explored to produce economically important species while preserving the genetic integrity of wild populations, as illustrated in the triploid infertile brown alga *Saccharina japonica* (kelp) with improved agronomic traits (102). This combination of economic advantages leveraged by increased biomass and lipid production combined with stress tolerance, an essential quality because of the inevitable stress conditions at industrial levels (100), and preservation of natural germplasm through infertility highlights the advantages of polyploid algal breeding.

Beyond biomass, algae are promising platforms to produce valuable pharmaceuticals (98). For example, the microalga *Haematococcus lacustris* synthesizes the antioxidant astaxanthin, which can reduce oxidative cellular stress 50 times more efficient than vitamin E. However, low natural yields have favored synthetic alternatives. However, induced polyploidization enhanced the production of astaxanthin by 60% at semi-industrial scale, paving the way for industrial production of natural astaxanthin in microalgae (103). Similar benefits were observed for polyploid *Nannochloropsis oculate,* which showed significantly higher biomass and pigment content upon colchicine treatment (104) and for polyploid *D. salina*, in which auxin and gibberellin treatments increased carotenoid and starch accumulation (101). Overall, using polyploidization as a “crop” improvement strategy in microalgae enhances their value as production systems for biofuels and high-value pharmaceutical components.

### White Bioeconomy.

#### Polyploid micro-organisms as industrial workhorses.

Microorganisms, alongside plants and aquatic organisms, form a third essential pilar of the bioeconomy transition. The white bioeconomy employs industrial biotechnology which uses living cells such as yeast, bacteria, or fungi, or their enzymes, to produce pharmaceuticals, materials, and energy, often with reduced energy and water use relative to conventional chemical processes (105). Nevertheless, most biomanufactured products are currently niche high-value products because production costs still exceed those of petroleum-based alternatives (106). Realizing the broader substitution potential of biomanufacturing therefore depends on continuous innovation and metabolic engineering of production strains to identify new products, suitable enzymes, and develop high-performing robust microorganisms capable of tolerating stressful process conditions in addition to high yields (106, 107).

As in other bioeconomies, polyploidization has been applied as a breeding tool in industrial yeast strains to obtain advanced stress tolerance and higher yields, for example, in lignocellulose-based bioethanol production (108, 109). Polyploid yeast outperformed diploid and haploid strains on corn hydrolysates, suggesting a positive relation between genome copy number and production (110). Attempts to create a superpolyploid yeast strain up to 32n further investigated this relationship and the genome stability of superpolyploids (109). Tetraploids also served as fertile intermediates to facilitate interspecific hybridization, resulting in phenotypes with distinct quality traits (111). Several methods now enable systematic hybridization of yeast strains across ploidy levels, like mata2-PBT (109), allowing the construction of an isogenic series of strains, and the iterative method of Hybrid Production to combine genomes of multiple ploidy yeast species (e.g., allododecaploids) with novel traits combinations relevant for brewing and industrial fermentation (112). These results illustrate how complex polyploid hybrids can generate emergent phenotypes that are difficult to obtain by conventional breeding, obtaining high-performing production strains adapted to stressful industrial conditions.

Bacteria offer a complementary source for bioproduct-producer strains. The alpha-proteobacterium *Zymomonas mobilis,* naturally converting sugars into ethanol, is identified as polyploid, parallel to industrial yeast in bioethanol production (113). Additionally, artificially inducing polyploidy in *Escherichia coli* improved the L-threonine production, further illustrating the impact of polyploidization on metabolic pathways in bacteria (114). Despite these advances, rapid and efficient strain design remains a bottleneck to obtaining biomanufactured products at competitive prices (106).

Polyploidization as driver for evolutionary innovation can be employed in the search for new innovative pathways with industrial applications. Where polyploidy slows evolution by masking effects of single mutations, it can accelerate neofunctionalization by providing redundant gene copies that diverge in a challenging environment to increase the fitness of cells (115). Polyploid cyanobacteria exemplify this potential through their striking morphological and ecological diversity compared to most other bacteria (115). Similarly, *Epulopiscium* bacteria, intestinal symbionts of the surgeonfish, adopted internal polyploidy as a strategy to maintain genetic diversity and sufficient recombination (116). Adaptive laboratory evolution (ALE) builds on these principles by forcing evolution under defined conditions to optimize strains for substrates or performance targets, facilitating the selection of desired phenotypes. ALE has been successfully used to identify interspecific yeast strains with improved fermentation traits or enhanced production of the carotenoid lycopene (117, 118). Interestingly, comparative analyses of Saccharomycotina species revealed that expansions of the same set of keystone gene families are associated with diverse metabolic phenotypes (119). As such knowledge accumulates, more targeted manipulations and screening for desired metabolic outcomes become feasible, positioning polyploidy and subsequent gene family expansion as a toolkit for evolving new metabolic capabilities in metabolic engineering.

Despite clear benefits, polyploidization is rarely tested in production strains other than yeast and *E. coli.* Given its long-standing success in crop improvement, as explained above, extending polyploid breeding to strain development might facilitate the development of the white bioeconomy toward a low-cost, high-volume products market. The intentions are similar: generating genetic diversity, optimizing yield, and generating robust cultures, preferably as quick and efficient as possible (106). However, a clear knowledge gap persists between academic discoveries and industrial implementation of those discoveries (105). Optimized knowledge transfer could enhance the benefits of white biotechnology production processes and thus the bioeconomy transition. Indeed, to transform available resources into a desired product, an in-depth understanding of the biological process is required before biological and bioprocess engineering can take place (107).

#### Polyploid bacteria for bioremediation.

As polyploidy can influence anabolic pathways to produce valuable products, it can also be leveraged to explore catabolic pathways to degrade toxic compounds, for example, in polluted water and soils, as part of environmental management strategies. As discussed earlier, additional genomic copies in bacteria and archaea can support growth under more extreme conditions such as deprivation of specific nutrient sources, giving them an evolutionary advantage (120). This stress adaptability is attractive to develop bioremediation strategies that are more ecologically friendly than the physiochemical-based methods (121). For example, polyploidy has been mentioned as a strategy for metabolic engineering in bioremediation of arsenic by microorganisms, while polyploid *Deinococcus radiodurans* is employed in uranium clean-up (121, 122). Additionally, investigation of microbial communities in systems mimicking crude-oil removal from cold seawater hinted toward the presence of polyploid *Pseudomonas,* inferred from differences in abundance across sequencing techniques (123). Supported by a mechanistic model in plants predicting polyploid establishment, even at small changes of metabolic efficiencies (124), one could hypothesize that polyploidization provides an advantage under polluted conditions and that microbial communities undergo a pollutant-driven metabolic shift at extreme environments. Natural extreme locations can thus serve as natural sinks to discover polyploid microorganisms with application potential, such as *Thermus thermophilus*, living at extreme temperatures (125). Harnessing resilience and pollutant-handling capacity in polyploid organisms can thus strengthen bioremediation approaches within sustainable environmental strategies.

### Red Bioeconomy.

#### Polyploid host cells for complex biologics.

Parallel to white biotechnology applications, altering ploidy states can boost the production of high-value, complex biopharmaceuticals. These biologics, including viral-vectors for cell and gene therapy, vaccines, and recombinant proteins, like insulin, require host cells capable of sophisticated modifications, typically provided by immortalized in vitro cell lines, such as Chinese Hamster Ovary cells (CHO) and HEK293 cells (126, 127). CHO cells are widely employed due to their fast adaptability to culture conditions, but this comes with the cost of high genomic instability and karyotypic diversity, which can jeopardize manufacturing consistency (128, 129). Interestingly, high-producing lines often show diverse phenotypes with spontaneous genomic rearrangements, copy number variations, and near-polyploid karyotypic variability (129, 130). Extra available genomic material can provide more transgenic insertion sites for transgenes and buffer disruptive insertion events, enabling sustained expression of recombinant products at levels that would be toxic or unstable in strictly diploid cells (131). However, further investigations that deliberate engineer ploidy are needed to uncover chromosome number-productivity and stability–adaptability tradeoffs (132). Near-tetraploid production cell lines are also intentionally generated by fusing mouse B cells encoding antibodies of interest with human myeloma cells, enabling efficient and unlimited monoclonal antibody production (133). This approach has revolutionized diagnostics and therapeutics, yet the inherent genomic instability of hybridomas, leading to off-target production and loss of specificity, underscores the need for continued innovation (134). Although many questions remain to stabilize such production lines, these examples illustrate how altering ploidy can optimize biopharmaceutical manufacturing and pave the way toward systemic host-cell engineering, in parallel to strain development in white biotechnology.

#### Ploidy shifts therapy resistance and therapies.

Adaptability and polyploidy-related stress resilience can also imply resistance to deliberately applied abiotic stress. In cancer, altered ploidy states have been implicated in recurrence and therapeutic resistance in response to an altered microenvironment initiated by therapeutics (135). Polyploid giant cancer cells (PGCCs), arising from genome doubling of a cancer cell and previously classified as nonfunctional due to its highly deformed cell shape and unstructured chromatin (136), enter cell cycle arrest upon therapeutic treatment. By being a nondividing cell, DNA integrity is protected from therapy-induced damage. Once the treatment ceases and microenvironmental stress diminishes, PGCCs can undergo depolyploidization and reenter the cell cycle, ensuring tumor survival (135). Targeting such PGCCs is an emerging strategy to reduce tumor resistance and disease relapses across cancer types (137). High-throughput screens have identified small molecules that interfere with the PGCC life cycle, indicate an arrest of PGCC formation, and can improve disease outcomes when combined with traditional therapies, supporting more personalized and precision medicine (138, 139). Despite encouraging results, drugs that specifically target therapy-associated polyploidization remain scares. Also, polyploidy has been linked to drugs resistance (115) and enhanced evolvability in pathogenic bacteria and human fungal pathogens (115, 140, 141). Morphological alterations of human fungal pathogens during pathogenesis are associated with ploidy differences, underscoring the broader relevance of ploidy dynamics in treatment responses. Thus, deeper understanding of underlying mechanisms stirring polyploidization could substantially improve therapeutic strategies.

Beyond cancer, polyploidy has been observed as a pathological state in diabetes and aging, with mechanistic Target of Rapamycin signaling proposed as a mechanistic link (142). In diabetic in vivo models, thyroid hormone administration normalized the DNA content and reversed the pathological state, suggesting a functional link between diabetes and polyploidy, and hinting at therapeutic benefits of targeting polyploidy in diabetes (143). Conversely, polyploidy is also recognized as a normal important physiological state in regeneration and tissue repair (144). Polyploid liver cells, for example, contribute to the organ’s metabolic capacity and regenerative plasticity through their increased genetic and metabolic diversity and their ability to undergo diploidization (145). Experimental manipulation of ploidy to enhance regenerative responses and tissue repair has shown promise in rodent models, but such interventions remain largely experimental due to unresolved safety concerns in clinical settings (145). The dual role of polyploidy, supporting adaptation and regeneration in healthy tissues, while enabling survival and relapses in malignant ones, complicates its therapeutic exploitation and highlights the need for integrative, multidisciplinary research to fully unlock all possibilities.

The resilience and adaptability of polyploid cells remain underexplored in medicine. Insights from plant and microbial systems suggest that polyploid-associated traits, stress tolerance, rapid evolution, and invasiveness, are directly relevant to cancers, infectious diseases, and resistant pathogens. Therefore, systematically incorporating ploidy dynamics in cancer biology, pathogen evolution, and disease treatment could reveal new vulnerabilities and hold promising steps forward to battle major global health threats.

## Conclusion

Polyploidization emerges as a shared mechanism, generating new trait combinations. Despite the diversity of organisms and applications, similar patterns arise: increased genetic diversity, expanded metabolic capacity, and altered morphology, often leading to enhanced tolerance to biotic and abiotic stresses. However, outcomes can be highly variable and range from increased performance and ecological success to genomic instability and maladaptation. Therefore, induced (natural or artificial) polyploidization should go hand-in-hand with careful phenotypic and ecological assessment. Polyploidy remains unevenly exploited across bioeconomy sectors, and risks are not always well characterized. Lessons from green biotechnology can help guide responsible innovation in blue, white, and red applications.

Recent bioeconomy definitions propose a dynamic concept, with more moderate economic growth expectations, and a more comprehensive social-ecological transformation integrating several disciplines such as molecular biology, biotechnology, ecology, and engineering, to balance economic growth with ecological considerations (146). Because polyploidization can reshape biological systems from cells to entire ecosystems in four biotechnology fields (3), the increasing exploitation of polyploidy holds considerable promise for accelerating the transition to a more sustainable and climate-smart bioeconomy.

## Supplementary Material

Appendix 01 (PDF)

## Data Availability

There are no data underlying this work.

## References

[1] P. S. Soltis, X. Liu, D. B. Marchant, C. J. Visger, D. E. Soltis, Polyploidy and novelty: Gottlieb’s legacy. Philos. Trans. R. Soc. Lond. B Biol. Sci. **369**, 20130351 (2014). 24958924 10.1098/rstb.2013.0351 PMC4071524

[2] L. Comai, The advantages and disadvantages of being polyploid. Nat. Rev. Genet. **6**, 836–846 (2005). 16304599 10.1038/nrg1711

[3] D. T. Fox, D. E. Soltis, P. S. Soltis, T. L. Ashman, Y. Van de Peer, Polyploidy: A biological force from cells to ecosystems. Trends Cell Biol. **30**, 688–694 (2020). 32646579 10.1016/j.tcb.2020.06.006 PMC7484144

[4] J. P. Morris, T. Baslan, D. E. Soltis, P. S. Soltis, D. T. Fox, Integrating the study of polyploidy across organisms, tissues, and disease. Annu. Rev. Genet. **22**, 46 (2025). 10.1146/annurev-genet-111523-102124 PMC11590481 39227132

[5] Y. Van De Peer, E. Mizrachi, K. Marchal, The evolutionary significance of polyploidy. Nat. Rev. Genet. **18**, 411–424 (2017). 28502977 10.1038/nrg.2017.26

[6] P. S. Soltis, D. E. Soltis, The role of hybridization in plant speciation. Annu. Rev. Plant Biol. **60**, 561–588 (2009). 19575590 10.1146/annurev.arplant.043008.092039

[7] J. H. Leebens-Mack , One thousand plant transcriptomes and the phylogenomics of green plants. Nature **574**, 679–685 (2019). 31645766 10.1038/s41586-019-1693-2 PMC6872490

[8] H. Chen, F. Almeida-Silva, G. Logghe, D. Bonte, Y. Van de Peer, The rise of polyploids during environmental catastrophes. bioRxiv [Preprint] (2024). 10.1101/2024.11.22.624806 (Accessed 11 June 2025). 42105759

[9] K. Bomblies, When everything changes at once: Finding a new normal after genome duplication: Evolutionary response to polyploidy. Proc. R. Soc. B **287**, 2020154 (2020). 10.1098/rspb.2020.2154 PMC7739491 33203329

[10] P. Baduel, S. Bray, M. Vallejo-Marin, F. Kolář, L. Yant, The “Polyploid Hop”: Shifting challenges and opportunities over the evolutionary lifespan of genome duplications. Front. Ecol. Evol. **6**, 117 (2018).

[11] P. P. Edger , Natural neopolyploids: A stimulus for novel research. New Phytol. **246**, 78–93 (2025). 39953679 10.1111/nph.20437 PMC11883059

[12] M. M. Bugge, T. Hansen, A. Klitkou, What is the bioeconomy? A review of the literature. Sustainability (Switzerland) **8**, 691 (2016).

[13] European Commission, A sustainable bioeconomy for Europe: Strengthening the connection between economy, society and the environment. COM(2018) 673 final, Brussels, (2018). https://eur-lex.europa.eu/legal-content/EN/TXT/PDF/?uri=CELEX:52018DC0673, (Accessed 21 July 2025).

[14] A. D. Twyford , The polyploid continuum and the landscape of polyploid genomic variation. Am. J. Bot. **112**, e70121 (2025). 41178014 10.1002/ajb2.70121 PMC12640477

[15] P. Kafarski, Rainbow code of biotechnology. CHEMIK **66**, 811–816 (2012).

[16] S. Rauf , Induced polyploidy: A tool for forage species improvement. Agriculture **11**, 210 (2021).

[17] J. Hufnagel, M. Reckling, F. Ewert, Diverse approaches to crop diversification in agricultural research. A review. Agron. Sustain. Dev. **40**, 14 (2020).

[18] A. K. E. Ekroth, C. Rafaluk-Mohr, K. C. King, Diversity and disease: evidence for the monoculture effect beyond agricultural systems. bioRxiv [Preprint] (2019). http://biorxiv.org/lookup/doi/10.1101/668228 (Accessed 16 July 2025). 10.1098/rspb.2019.1811 PMC6774778 31551053

[19] A. S. Krug, E. B. M. Drummond, D. L. Van Tassel, E. J. Warschefsky, The next era of crop domestication starts now. Proc. Natl. Acad. Sci. U.S.A. **120**, e2205769120 (2023). 36972445 10.1073/pnas.2205769120 PMC10083606

[20] Y. Niu , Phenotypic advantages and improved genomic stability following selection in advanced selfing-generations of *Brassica* allohexaploids. J. Genet. Genomics **52**, 799–811 (2025). 40090574 10.1016/j.jgg.2025.03.004

[21] P. Zou , In vitro induction of tetraploids in the ornamental plant Melastoma candidum D. Don using colchicine treatment. In Vitro Cell Dev Biol Plant. **61**, 789–801 (2025).

[22] M. Ochiai, K. Okuda, Y. Kawahara, Y. Matsumoto, H. Fukui, “Polyploidy induction by colchicine treatment in kenaf (Hibiscus cannabinus L.)” in Combined Proceedings IPPS **74**, (2024), pp. 28–33.

[23] Y. Xu , Polyploid crape myrtle was produced through hybridizing with 2n gametes induced by high-temperature. Euphytica **221**, 30 (2025).

[24] F. Kauai , Interspecific transfer of genetic information through polyploid bridges. Proc. Natl. Acad. Sci. U.S.A. **121**, e2400018121 (2024). 38748576 10.1073/pnas.2400018121 PMC11126971

[25] L. Verleysen , Characterization of the genetic composition and establishment of a core collection for the INERA Robusta coffee (*Coffea canephora*) field genebank from the Democratic Republic of Congo. Front. Sustain. Food Syst. **7**, 1239442 (2023).

[26] H. H. Tai, L. M. Shannon, M. V. Strömvik, Polyploidy in potatoes: Challenges and possibilities for climate resilience. Trends Genet. **41**, 716–723 (2025). 40268598 10.1016/j.tig.2025.03.006

[27] R. Ortiz, S. J. Peloquin, Effect of sporophytic heterozygosity on the male gametophyte of the tetraploid potato. Ann. Bot. **73**, 61–64 (1994).

[28] P. Du , Development and characterization of bacterial wilt-resistant synthetic polyploid peanuts. Crop J. **13**, 125–134 (2025).

[29] O. Shelef, P. J. Weisberg, F. D. Provenza, The value of native plants and local production in an era of global agriculture. Front. Plant Sci. **8**, 2069 (2017). 29259614 10.3389/fpls.2017.02069 PMC5723411

[30] Y. Dussert , Recurrent evolution of cryptic triploids in cultivated enset increases yield. bioRxiv [Preprint] (2025). http://biorxiv.org/lookup/doi/10.1101/2025.02.14.638218 (Accessed 8 July 2025). 10.1371/journal.pgen.1012241 PMC13426944 42497221

[31] Y. Qu, A. R. Fernie, J. Liu, J. Yan, Doubled haploid technology and synthetic apomixis: Recent advances and applications in future crop breeding. Mol. Plant **17**, 1005–1018 (2024). 38877700 10.1016/j.molp.2024.06.005

[32] H. Ceballos, R. S. Kawuki, V. E. Gracen, G. C. Yencho, C. H. Hershey, Conventional breeding, marker-assisted selection, genomic selection and inbreeding in clonally propagated crops: A case study for cassava. Theor. Appl. Genet. **128**, 1647–1667 (2015). 26093610 10.1007/s00122-015-2555-4 PMC4540783

[33] C. Liu , Exceptionally high genetic variance of the doubled haploid (DH) population of poplar. J. For. Res. (Harbin) **34**, 1941–1950 (2023).

[34] D. Kuniyoshi, Y. Kishima, Fertile interspecific diploid hybrids between the Asian and African rice species facilitated by tetraploidization and its reduction. Theor. Appl. Genet. **138**, 161 (2025). 40576682 10.1007/s00122-025-04901-3 PMC12204913

[35] B. Hale, A. M. R. Ferrie, S. Chellamma, J. P. Samuel, G. C. Phillips, Androgenesis-based doubled haploidy: Past, present, and future perspectives. Front. Plant Sci. **12**, 751230 (2022). 35069615 10.3389/fpls.2021.751230 PMC8777211

[36] J. Böhm, W. Schipprack, H. F. Utz, A. E. Melchinger, Tapping the genetic diversity of landraces in allogamous crops with doubled haploid lines: A case study from European flint maize. Theor. Appl. Genet. **130**, 861–873 (2017). 28194473 10.1007/s00122-017-2856-x

[37] J. K. Yu, Y. S. Chung, Plant variety protection: Current practices and insights. Genes (Basel) **12**, 1127 (2021). 34440301 10.3390/genes12081127 PMC8392850

[38] C. J. Yang , Overcoming barriers to the registration of new plant varieties under the DUS system. Commun. Biol. **4**, 302 (2021). 33686157 10.1038/s42003-021-01840-9 PMC7940638

[39] C. E. R. Lehmann , Functional diversification enabled grassy biomes to fill global climate space. bioRxiv [Preprint] (2019). http://biorxiv.org/lookup/doi/10.1101/583625 (Accessed 18 July 2025).

[40] M. C. Stitzer , Extensive genome evolution distinguishes maize within a stable tribe of grasses. bioRxiv [Preprint] (2025). http://biorxiv.org/lookup/doi/10.1101/2025.01.22.633974 (Accessed 8 July 2025).

[41] G. Batiru, T. Lübberstedt, Polyploidy in maize: From evolution to breeding. Theor. Appl. Genet. **137**, 182 (2024). 39001883 10.1007/s00122-024-04688-9

[42] N. U. Ain , Genetic determinants of biomass in C4 crops: Molecular and agronomic approaches to increase biomass for biofuels. Front. Plant Sci. **13**, 839588 (2022). 35812976 10.3389/fpls.2022.839588 PMC9260593

[43] M. Basili, M. A. Rossi, *Brassica carinata*-derived biodiesel production: Economics, sustainability and policies. The Italian case. J. Clean. Prod. **191**, 40–47 (2018).

[44] O. V. Melnychuk , The technology used for synthetic polyploid production of *Miscanthus* as cellulosic biofuel feedstock. Open Agric. J. **14**, 164–173 (2020).

[45] M. J. Serapiglia , Ploidy level affects important biomass traits of novel shrub willow (*Salix*) hybrids. Bioenergy Res. **8**, 259–269 (2015).

[46] S. Corneillie , Polyploidy affects plant growth and alters cell wall composition. Plant Physiol. **179**, 74–87 (2019). 30301776 10.1104/pp.18.00967 PMC6324247

[47] M. Madadi , Modified lignocellulose and rich starch for complete saccharification to maximize bioethanol in distinct polyploidy potato straw. Carbohydr. Polym. **265**, 118070 (2021). 33966834 10.1016/j.carbpol.2021.118070

[48] N. V. Hoang, A. Furtado, F. C. Botha, B. A. Simmons, R. J. Henry, Potential for genetic improvement of sugarcane as a source of biomass for biofuels. Front. Bioeng. Biotechnol. **3**, 182 (2015). 26636072 10.3389/fbioe.2015.00182 PMC4646955

[49] R. Seepaul , *Brassica carinata*: Biology and agronomy as a biofuel crop. GCB Bioenergy **13**, 582–599 (2021).

[50] C. Dinu, G. Cican, S. Osman, R. Secareanu, Performance and emissions of camelina biodiesel-Jet A blends in a micro-gas turbine as a sustainable pathway for aviation. Fire **8**, 442 (2025).

[51] L. Dominguez Mendez, A. J. Studer, Is more better? Polyploidy in crops with diverse end uses and the potential for future applications. Ann. Bot. **137**, mcaf253 (2025). 10.1093/aob/mcaf253 PMC12933660 41132109

[52] S. Kumar, “Impact of ploidy changes on secondary metabolites productions in plants” in Evolutionary Diversity as a Source for Anticancer Molecules, A. K. Srivastava, V. K. Kannaujiya, R. K. Singh, D. Singh, Eds. (Elsevier Science Ltd., 2020), pp. 29–46.

[53] H. Madani , Effect of polyploidy induction on natural metabolite production in medicinal plants. Biomolecules **11**, 899 (2021). 34204200 10.3390/biom11060899 PMC8234191

[54] J. K. Weng, R. N. Philippe, J. P. Noel, The rise of chemodiversity in plants. Science **1979**, 1667–1670 (2012). 10.1126/science.1217411 22745420

[55] C. Godswill, Medicinal plants: The medical, food, and nutritional biochemistry and uses. Int. J. Adv. Acad. Res. **5**, 2488–9849 (2019).

[56] A. Martínez-Aguilar , Tilianin content and morphological characterization of colchicine-induced autotetraploids in *Agastache mexicana*. PeerJ **12**, e18545 (2024). 39588001 10.7717/peerj.18545 PMC11587875

[57] P. Hongbing , Induction and evaluation of autotetraploid pomegranate. J. Henan Agric. Sci. **49**, 111–117 (2020).

[58] J.-Y. Xue , Multi-omics roadmap to plant-derived medicines. Engineering. 10.1016/j.eng.2025.10.019 (2025).

[59] W. Su , Polyploidy underlies co-option and diversification of biosynthetic triterpene pathways in the apple tribe. Proc. Natl. Acad. Sci. U.S.A. **118**, e2101767118 (2021). 33986115 10.1073/pnas.2101767118 PMC8157987

[60] S. H. Hamarashid, Y. Khaledian, F. Soleimani, In vitro polyploidy-mediated enhancement of secondary metabolites content in *Stachys byzantina* L. Genet. Resour. Crop Evol. **69**, 719–728 (2022).

[61] Y. van de Peer, T. L. Ashman, P. S. Soltis, D. E. Soltis, Polyploidy: An evolutionary and ecological force in stressful times. Plant Cell **33**, 11–26 (2021). 33751096 10.1093/plcell/koaa015 PMC8136868

[62] M. Ebadi , The duplication of genomes and genetic networks and its potential for evolutionary adaptation and survival during environmental turmoil. Proc. Natl. Acad. Sci. U.S.A. **120**, e2307289120 (2023). 37788315 10.1073/pnas.2307289120 PMC10576144

[63] G. S. Malhi, M. Kaur, P. Kaushik, Impact of climate change on agriculture and its mitigation strategies: A review. Sustainability **13**, 1318 (2021).

[64] S. O. Oshunsanya, N. J. Nwosu, Y. Li, “Abiotic stress in agricultural crops under climatic conditions” in Sustainable Agriculture, Forest and Environmental Management, M. K. Jhariya, A. Banerjee, R. S. Meena, D. K. Yadav, Eds. (Springer Singapore, 2019), pp. 71–100.

[65] H. Chen , Induction of new tetraploid genotypes and heat tolerance assessment in *Asparagus officinalis* L. Sci. Hortic. **264**, 109168 (2020).

[66] S. Liu , In vitro induced tetraploid of *Dendranthema nankingense* (Nakai) Tzvel. shows an improved level of abiotic stress tolerance. Sci. Hortic. **127**, 411–419 (2011).

[67] V. Kemesyte, G. Statkeviciute, G. Brazauskas, Perennial ryegrass yield performance under abiotic stress. Crop Sci. **57**, 1935–1940 (2017).

[68] L. Wang , DNA hypomethylation in tetraploid rice potentiates stress-responsive gene expression for salt tolerance. Proc. Natl. Acad. Sci. U.S.A. **118**, e2023981118 (2021). 33771925 10.1073/pnas.2023981118 PMC8020803

[69] R. Li , Deciphering salt stress adaptation in octoploid broomcorn millet (*Panicum miliaceum* L.): For sustainable agricultural development in saline-alkaline soils. J. Environ. Manage. **387**, 125713 (2025). 40403670 10.1016/j.jenvman.2025.125713

[70] A. Ræbild , Polyploidy—A tool in adapting trees to future climate changes? A review of polyploidy in trees. For. Ecol. Manage. **560**, 121767 (2024).

[71] M. Eisenring , Genotypic variation rather than ploidy level determines functional trait expression in a foundation tree species in the presence and absence of environmental stress. Ann. Bot. **131**, 229–242 (2023). 35641114 10.1093/aob/mcac071 PMC9904343

[72] A. Hassani, A. Azapagic, N. Shokri, Global predictions of primary soil salinization under changing climate in the 21st century. Nat. Commun. **12**, 6663 (2021). 34795219 10.1038/s41467-021-26907-3 PMC8602669

[73] Y. Yuan , Proso millet (*Panicum miliaceum* L.): A potential crop to meet demand scenario for sustainable saline agriculture. J. Environ. Manage. **296**, 113216 (2021). 34237674 10.1016/j.jenvman.2021.113216

[74] Y. Wang , Genomic and evolutionary evidence for drought adaptation of grass allopolyploid *Brachypodium hybridum*. J. Exp. Bot. **76**, 2924–2938 (2025). 40102696 10.1093/jxb/eraf128 PMC12223499

[75] G. Wang, X. Li, X. Xi, W. F. Cong, Crop diversification reinforces soil microbiome functions and soil health. Plant Soil **476**, 375–383 (2022).

[76] T. L. Ashman, Uncovering the reciprocal effects of plant polyploidy and the microbiome: Implications for understanding of polyploid success. New Phytol. **247**, 1060–1070 (2025). 40432236 10.1111/nph.70226 PMC12222933

[77] H. M. L. Wipf, D. Coleman-Derr, Evaluating domestication and ploidy effects on the assembly of the wheat bacterial microbiome. PLoS ONE **16**, e0248030 (2021). 33735198 10.1371/journal.pone.0248030 PMC7971525

[78] A. Thébault, B. Frey, E. A. D. Mitchell, A. Buttler, Species-specific effects of polyploidisation and plant traits of *Centaurea maculosa* and *Senecio inaequidens* on rhizosphere microorganisms. Oecologia **163**, 1011–1020 (2010). 20229242 10.1007/s00442-010-1598-0

[79] Y. Zhou, A. Stepanenko, O. Kishchenko, J. Xu, N. Borisjuk, Duckweeds for phytoremediation of polluted water. Plants **12**, 589 (2023). 36771672 10.3390/plants12030589 PMC9919746

[80] M. M. Turcotte, N. Kaufmann, K. L. Wagner, T. A. Zallek, T. L. Ashman, Neopolyploidy increases stress tolerance and reduces fitness plasticity across multiple urban pollutants: Support for the “general-purpose” genotype hypothesis. Evol. Lett. **8**, 416–426 (2024). 38818423 10.1093/evlett/qrad072 PMC11134461

[81] Q. Bafort, T. Wu, A. Natran, O. De Clerck, Y. Van De Peer, The immediate effects of polyploidization of *Spirodela polyrhiza* change in a strain-specific way along environmental gradients. Evol. Lett. **7**, 37–47 (2023). 37065435 10.1093/evlett/qrac003 PMC10091501

[82] I. Ben Said, A. Muscolo, I. Mezghani, M. Chaieb, Treated municipal wastewater as option to the use of fresh water for the cultivation of valuable pastoral species Buffel grass (*Cenchrus ciliaris* L.). Water Environ. J. **37**, 549–560 (2023).

[83] M. Te Beest , The more the better? The role of polyploidy in facilitating plant invasions. Ann. Bot. **109**, 19–45 (2012). 22040744 10.1093/aob/mcr277 PMC3241594

[84] S. J. Yang , Divergence in cold tolerance promotes niche differentiation between diploid and polyploid kiwifruits along an altitudinal gradient in Southwest China. Oikos **2024**, e10181 (2024).

[85] F. Mortier , Understanding polyploid establishment: Temporary persistence or stable coexistence? Oikos **2024**, e09929 (2024).

[86] S. Keyvanshokooh, The performance of triploids versus diploids in aquaculture: A review through the omics window. Aquacult. Int. **33**, 36 (2025).

[87] L. Chen, J. Xu, X. Sun, P. Xu, Research advances and future perspectives of genomics and genetic improvement in allotetraploid common carp. Rev. Aquac. **14**, 957–978 (2022).

[88] J. L. Everson, G. M. Weber, M. L. Manor, J. C. Tou, P. B. Kenney, Polyploidy affects fillet yield, composition, and fatty acid profile in two-year old, female rainbow trout. *Oncorhynchus mykiss*. Aquaculture **531**, 735873 (2021).

[89] W. Wan , Genetic improvement of aquaculture performance for tetraploid Pacific oysters, *Crassostrea gigas*: A case study of four consecutive generations of selective breeding. Aquaculture **563**, 738910 (2023).

[90] C. S. Ribeiro , The effect of ploidy on the fatty acid profile during the reproductive cycle of female rainbow trout (*Oncorhynchus mykiss*). Aquacult. Int. **20**, 1117–1137 (2012).

[91] T. J. Benfey, Effectiveness of triploidy as a management tool for reproductive containment of farmed fish: Atlantic salmon (*Salmo salar*) as a case study. Rev. Aquac. **8**, 264–282 (2016).

[92] A. Delaval , Chromosomal aberrations and early mortality in a non-mammalian vertebrate: Example from pressure-induced triploid Atlantic salmon. Heredity (Edinb.). **133**, 426–436 (2024). 39369146 10.1038/s41437-024-00727-9 PMC11589116

[93] R. F. Lincoln, A. P. Scott, Production of all-female triploid rainbow trout. Aquaculture **30**, 375–380 (1983).

[94] R. S. Rasmussen, M. T. Morrissey, Biotechnology in aquaculture: Transgenics and polyploidy. Compr. Rev. Food Sci. Food Saf. **6**, 2–16 (2007).

[95] T. Kon , The genetic basis of morphological diversity in domesticated goldfish. Curr. Biol. **30**, 2260.e6–2274.e6 (2020). 32392470 10.1016/j.cub.2020.04.034

[96] I. Braasch, Genome evolution: Domestication of the allopolyploid Goldfish. Curr. Biol. **30**, R812–R815 (2020). 32693075 10.1016/j.cub.2020.05.073

[97] B. Liu , The first genome-wide survey analysis of the Tibetan Plateau tetraploid *Schizothorax curvilabiatus* reveals its microsatellite characteristics and phylogenetic relationships. Genes (Basel) **16**, 491 (2025). 40428313 10.3390/genes16050491 PMC12111650

[98] D. Y. Y. Tang, C. Ooi, P. L. Show, Blue bioeconomy and biotechnology: Towards a sustainably growing microalgae industry. Blue Biotechnol. **2**, 13 (2025).

[99] A. Rock, L. Novoveská, D. Green, Synthetic biology is essential to unlock commercial biofuel production through hyper lipid-producing microalgae: A review. Appl. Phycol. **2**, 41–59 (2021).

[100] M. Kwak , Improvement of biomass and lipid yield under stress conditions by using diploid strains of *Chlamydomonas reinhardtii*. Algal Res. **26**, 180–189 (2017).

[101] H. Mansouri, F. S. Nezhad, Changes in growth and biochemical parameters in *dunaliella saline* (Dunaliellaceae) in response to auxin and gibberellin under colchicine-induced polyploidy. J. Phycol. **57**, 1284–1294 (2021). 33817802 10.1111/jpy.13173

[102] P. Tian , Polyploid breeding of *Saccharina japonica*: Harnessing aposporous reproduction. Aquaculture **604**, 742491 (2025).

[103] R. Le-Feuvre , Chemical induction of polyploidy increases astaxanthin accumulation capacity in the microalgae *Haematococcus lacustris* (Gir.-Chantr.) Rostaf. Algal Res. **59**, 102465 (2021).

[104] K. Rahmawati, Y. Yunianta, Y. Risjani, Pigment production of Nannochloropsis oculata (eustigmatophyceae) under different colchicine concentration. Biotechnol. Biotechnol. Equip. **38**, 1 (2024). 10.21203/rs.3.rs-2493793/v1.

[105] M. Kordi , White biotechnology and the production of bio-products. Systems Microbiology and Biomanufacturing **2**, 413–429 (2022).

[106] E. Abbate , Optimizing the strain engineering process for industrial-scale production of bio-based molecules. J. Ind. Microbiol. Biotechnol. **50**, 1 (2023). 10.1093/jimb/kuad025 PMC10548853 37656881

[107] A. J. J. Straathof , Grand research challenges for sustainable industrial biotechnology. Trends Biotechnol. **37**, 1042–1050 (2019). 31054854 10.1016/j.tibtech.2019.04.002

[108] S. Mozzachiodi, K. Krogerus, B. Gibson, A. Nicolas, G. Liti, Unlocking the functional potential of polyploid yeasts. Nat. Commun. **13**, 2580 (2022). 35545616 10.1038/s41467-022-30221-x PMC9095626

[109] S. Hirota, Y. Nakayama, K. Ekino, S. Harashima, Highly genomic instability of super-polyploid strains of *Saccharomyces cerevisiae*. J. Biosci. Bioeng. **137**, 77–84 (2024). 38135639 10.1016/j.jbiosc.2023.11.009

[110] L. Liu , Engineered polyploid yeast strains enable efficient xylose utilization and ethanol production in corn hydrolysates. Front. Bioeng. Biotechnol. **9**, 655272 (2021). 33748094 10.3389/fbioe.2021.655272 PMC7973232

[111] S. Naseeb , Restoring fertility in yeast hybrids: Breeding and quantitative genetics of beneficial traits. Proc. Natl. Acad. Sci. U.S.A. **118**, e2101242118 (2021). 34518218 10.1073/pnas.2101242118 PMC8463882

[112] D. Peris , Synthetic hybrids of six yeast species. Nat. Commun. **11**, 2085 (2020). 32350251 10.1038/s41467-020-15559-4 PMC7190663

[113] K. Fuchino, D. Wasser, J. Soppa, Genome copy number quantification revealed that the ethanologenic Alpha-proteobacterium *Zymomonas mobilis* is polyploid. Front. Microbiol. **12**, 705895 (2021). 34408736 10.3389/fmicb.2021.705895 PMC8365228

[114] S. Wang , Creating polyploid *Escherichia coli* and its application in efficient L-threonine production. Adv. Sci. **10**, 2302417 (2023). 10.1002/advs.202302417 PMC10625114 37749873

[115] T. S. Hatakeyama, R. Ohbayashi, Evolutionary Innovation by Polyploidy. PRX Life **2**, 043021 (2024).

[116] E. R. Angert, Challenges faced by highly polyploid bacteria with limits on DNA inheritance. Genome Biol. Evol. **13**, 6 (2021). 10.1093/gbe/evab037 PMC8245194 33677487

[117] D. Peris , Hybridization and adaptive evolution of diverse *Saccharomyces* species for cellulosic biofuel production. Biotechnol. Biofuels **10**, 78 (2017). 28360936 10.1186/s13068-017-0763-7 PMC5369230

[118] K. Zhou , Adaptive evolution and metabolic engineering boost lycopene production in *Saccharomyces cerevisiae* via enhanced precursors supply and utilization. J. Agric. Food Chem. **71**, 3821–3831 (2023). 36802623 10.1021/acs.jafc.2c08579

[119] K. T. David , Convergent expansions of keystone gene families drive metabolic innovation in Saccharomycotina yeasts. Proc. Natl. Acad. Sci. U.S.A. **122**, e2500165122 (2025). 40460114 10.1073/pnas.2500165122 PMC12167968

[120] M. G. Naitam, M. Grover, R. Kaushik, Polyploidy in prokaryotes: Evolutionary advantages and strategy for survival under extreme conditions. Haya Saudi J. Life Sci. **6**, 205–212 (2021).

[121] A. Khan , The sustainable approach of microbial bioremediation of arsenic: An updated overview. Int. J. Environ. Sci. Technol. **21**, 7849–7864 (2024).

[122] T. Manobala, S. K. Shukla, T. Subba Rao, M. Dharmendira Kumar, A new uranium bioremediation approach using radio-tolerant *Deinococcus radiodurans* biofilm. J. Biosci. **44**, 122 (2019). 31719231

[123] H. Nõlvak , Microbial community dynamics during biodegradation of crude oil and its response to biostimulation in Svalbard seawater at low temperature. Microorganisms **9**, 2425 (2021). 34946026 10.3390/microorganisms9122425 PMC8707851

[124] S. Milosavljevic, F. Kauai, F. Mortier, Y. Van Peer, D. Bonte, A metabolic perspective on polyploid invasion and the emergence of life histories: Insights from a mechanistic model. Am. J. Bot. **111**, e16387 (2024). 39113228 10.1002/ajb2.16387 PMC7616395

[125] N. Ohtani, M. Tomita, M. Itaya, An extreme thermophile, *Thermus thermophilus*, is a polyploid bacterium. J. Bacteriol. **192**, 5499–5505 (2010). 20729360 10.1128/JB.00662-10 PMC2950507

[126] L. Cordova, K. Lee, Cells as a biofactory: Parallels between biopharmaceutical manufacturing and industrial biotechnology. Industrial Biotechnology **19**, 208–217 (2023).

[127] G. Smesnik, N. Virgolini, M. Toth, A. Dürauer, N. Borth, Comparative analysis of HEK293 genomic variability. Biotechnol. Bioeng. **123**, 436–448 (2025). 41211952 10.1002/bit.70105 PMC12779178

[128] M. J. Wurm, F. M. Wurm, Naming CHO cells for bio-manufacturing: Genome plasticity and variant phenotypes of cell populations in bioreactors question the relevance of old names. Biotechnol. J. **16**, 2100165 (2021). 10.1002/biot.202100165 34050613

[129] S. Huhn , Chromosomal instability drives convergent and divergent evolution toward advantageous inherited traits in mammalian CHO bioproduction lineages. iScience **25**, 104074 (2022). 35355517 10.1016/j.isci.2022.104074 PMC8958363

[130] K. L. Syddall , Directed evolution of biomass intensive CHO cells by adaptation to sub-physiological temperature. Metab. Eng. **81**, 53–69 (2024). 38007176 10.1016/j.ymben.2023.11.005

[131] N. Yamano , Increased recombinant protein production owing to expanded opportunities for vector integration in high chromosome number Chinese hamster ovary cells. J. Biosci. Bioeng. **122**, 226–231 (2016). 26850366 10.1016/j.jbiosc.2016.01.002

[132] N. Yamano-Adachi, H. Hata, Y. Nakanishi, T. Omasa, Effects of genome instability of parental CHO cell clones on chromosome number distribution and recombinant protein production in parent-derived subclones. J. Biosci. Bioeng. **137**, 54–63 (2024). 37981489 10.1016/j.jbiosc.2023.10.001

[133] S. Dübel, Can antibodies be “vegan”? A guide through the maze of today’s antibody generation methods. MAbs **16**, 2343499 (2024). 38634488 10.1080/19420862.2024.2343499 PMC11028021

[134] A. R. M. Bradbury , When monoclonal antibodies are not monospecific: Hybridomas frequently express additional functional variable regions. MAbs **10**, 539–546 (2018). 29485921 10.1080/19420862.2018.1445456 PMC5973764

[135] K. J. Pienta, E. U. Hammarlund, J. S. Brown, S. R. Amend, R. M. Axelrod, Cancer recurrence and lethality are enabled by enhanced survival and reversible cell cycle arrest of polyaneuploid cells. Proc. Natl. Acad. Sci. U.S.A. **118**, e2020838118 (2021). 33504594 10.1073/pnas.2020838118 PMC7896294

[136] S. R. Amend , Polyploid giant cancer cells: Unrecognized actuators of tumorigenesis, metastasis, and resistance. Prostate **79**, 1489–1497 (2019). 31376205 10.1002/pros.23877 PMC6706309

[137] Y. Ogawa, L. Fisher, T. Matsumoto, The impact of polyploid giant cancer cells: The root of stress resilience. Cancer Sci. **116**, 2949–2958 (2025). 40921651 10.1111/cas.70191 PMC12580870

[138] Y. Ma , High-throughput empirical and virtual screening to discover novel inhibitors of polyploid giant cancer cells in breast cancer. Anal. Chem. **97**, 5498–5506 (2025). 40040372 10.1021/acs.analchem.4c05138 PMC11923954

[139] X. Zhang , Targeting polyploid giant cancer cells potentiates a therapeutic response and overcomes resistance to PARP inhibitors in ovarian cancer. Sci. Adv. **9**, eadf7195 (2023). 37478190 10.1126/sciadv.adf7195 PMC10361597

[140] L. Sun , Effective polyploidy causes phenotypic delay and influences bacterial evolvability. PLoS Biol. **16**, e2004644 (2018). 29470493 10.1371/journal.pbio.2004644 PMC5839593

[141] Z. Li, K. Nielsen, Morphology changes in human fungal pathogens upon interaction with the host. J. Fungi **3**, 66 (2017). 10.3390/jof3040066 PMC5753168 29333431

[142] D. Choudhury , Polyploidy and mTOR signaling: A possible molecular link. Cell Commun. Signal. **22**, 196 (2024). 38539200 10.1186/s12964-024-01526-9 PMC10976710

[143] A. M. Lavecchia , Thyroid hormone treatment counteracts cellular phenotypical remodeling in diabetic organs. iScience **26**, 107826 (2023). 37752946 10.1016/j.isci.2023.107826 PMC10518716

[144] E. C. Bailey, S. Kobielski, J. Park, V. P. Losick, Polyploidy in tissue repair and regeneration. Cold Spring Harb. Perspect. Biol. **13**, a040881 (2021). 34187807 10.1101/cshperspect.a040881 PMC8485745

[145] S. Zare Jalise , Liver regeneration: Polyploidy and cellular senescence as potential regulators. Stem Cell Rev. Rep. **22**, 275–295 (2025). 41171586 10.1007/s12015-025-11001-8

[146] D. Eversberg, J. Holz, L. Pungas, The bioeconomy and its untenable growth promises: Reality checks from research. Sustain. Sci. **18**, 569–582 (2023).

