# Supplementary material for: Polyploidy: A macromutational force pushing bioeconomic developments
Source: Proc Natl Acad Sci U S A. 2026 May 26;123(22):e2522065123. doi: 10.1073/pnas.2522065123 (PMC13229275; doi:10.1073/pnas.2522065123)
Supplement: Supplementary file 1 — Appendix 01 (PDF) [file pnas.2522065123.sapp.pdf]

Supplementary materials

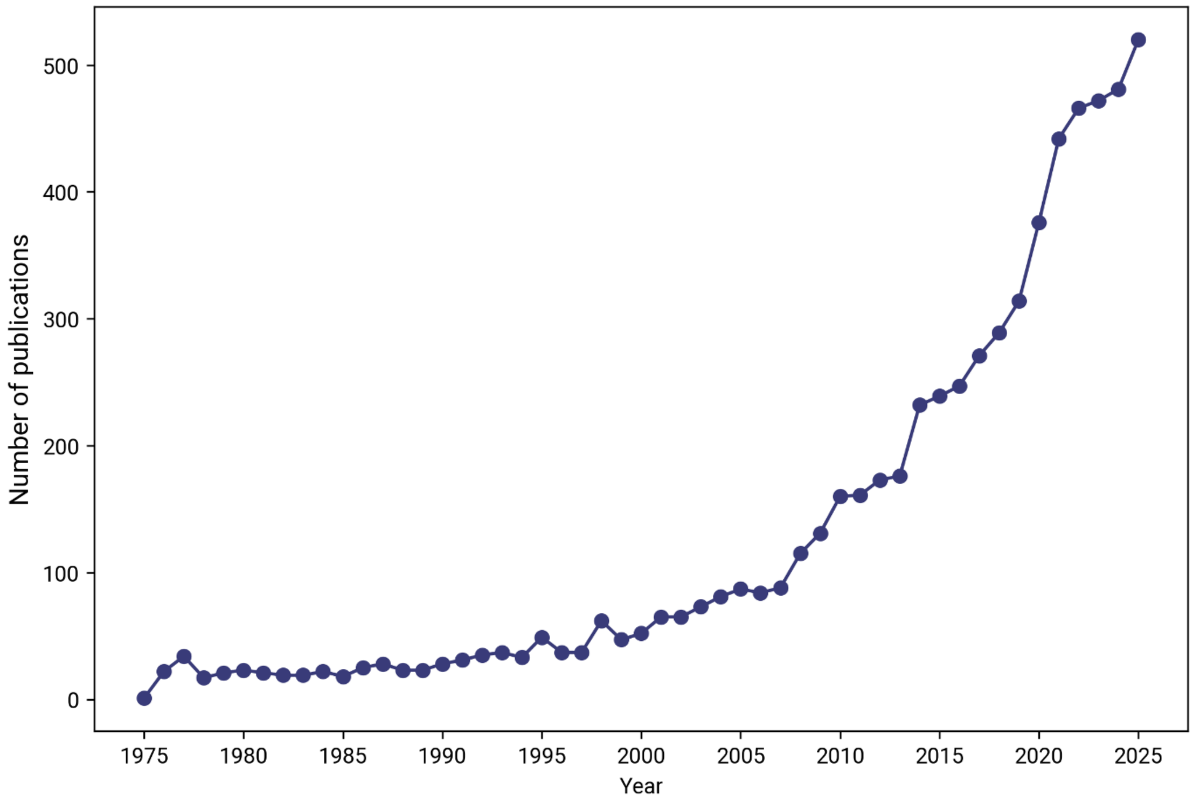

Figure S1. Evolution of the number of scientific papers published (until November 2025) on polyploidy/WGD within the different biotechnology sectors**.** The figure represents the number of unique research and review articles published from 1975 onwards (N = 6572) downloaded from Web of Sciences via the SmartSearch tool by searching for polyploidy and whole genome duplication (visited on 09/12/2025).
